# 206,977 newborn screening results reveal the ethnic differences in the spectrum of inborn errors of metabolism in Huaihua, China

**DOI:** 10.3389/fgene.2024.1387423

**Published:** 2024-05-09

**Authors:** Gang Xiao, Zonghui Feng, Chaochao Xu, Xuzhen Huang, Maosheng Chen, Min Zhao, Yanbin Li, Yang Gao, Shulin Wu, Yuyan Shen, Ying Peng

**Affiliations:** ^1^ Neonatal Disease Screening Center, Huaihua City Maternal and Child Health Care Hospital, Huaihua, Hunan Province, China; ^2^ Technical Support Center, Zhejiang Biosan Biochemical Technologies Co., Ltd, Hangzhou, Zhejiang Province, China; ^3^ Department of Medical Genetics, National Health Commission Key Laboratory of Birth Defects Research, Hunan Provincial Maternal and Child Healthcare Hospital, Changsha, China

**Keywords:** inborn errors of metabolism, newborn screening, incidence, ethnic group, ethnic features variants

## Abstract

**Background:**

Inborn errors of metabolism (IEMs) are rare diseases caused by inherited defects in various biochemical pathways that strongly correlate with early neonatal mortality and stunting. Currently, no studies have reported on the incidence of IEMs of multi-ethnic groups in Huaihua, China.

**Methods:**

A total of 206,977 neonates with self-reported ethnicity who underwent IEM screening at Huaihua from 2015 to 2021 were selected for observation. Among them, 69 suspected IEM-positive neonates were referred for urine gas chromatography-mass spectrometry analysis, biochemical detection, next-generation sequencing, and Sanger sequencing.

**Results:**

Sixty-nine newborns were diagnosed with IEMs, with an overall incidence of 1:3,000. The two most common disorders were 2-methylbutyryl glycinuria (1:7,137) and phenylalanine hydroxylase deficiency (1:22,997). Moreover, the incidence of IEMs in the minority ethnic group (Miao, Dong, Tujia and Yao) (1:1,852) was markedly higher than in the Han ethnic group (1:4,741). Some ethnic features variants were identified; NM_001609.4:c.1165A>G in the *ACADSB* gene for Miao and Dong ethnic groups, NM_014251.2:c.852_855del in the *SLC25A13* gene for Miao ethnic groups.

**Conclusion:**

This study revealed the IEM incidence within the minority ethnic groups is markedly higher than among the Han nationality and the gene variant spectrum is dramatically different in Huaihua, China. Hence, It serves as a theoretical reference for the screening and diagnosing of neonatal IEMs of multi-ethnic groups in the Huaihua area, and across China.

## 1 Introduction

Inborn errors of metabolism (IEMs) are a group of disorders that result from insufficient activity in the intermediary metabolism pathway (Therrell et al., 2015). Sir Archibald first coined the term “inborn errors of metabolism” in the early 19th century; to date, over 1,400 IEMs have been described (Mak et al., 2013). IEMs can present at any age and impact myriad cell types, tissues, and organs (Mak et al., 2013). However, serious consequences, including severe irreversible intellectual disability, epilepsy, metabolic acidosis, multiple organ damage, and premature death, can be prevented if early diagnosis and treatment are implemented following newborn screening for IEMs (Therrell et al., 2015; Zhang et al., 2021).

Newborn Screening (NBS) is a valuable method for detecting IEMs within the first few days of life and can, thus, diminish morbidity and mortality in early childhood, while also preventing severe health problems (Howson et al., 2018). NBS was first implemented in 1963 and has since developed into a comprehensive system capable of screening 40–50 disorders using a single blood sample (Fernhoff, 2009; Howson et al., 2018; Loeber et al., 2021). In particular, tandem mass spectrometry (MS/MS) facilitates the sensitive and specific identification of a wide range of IEMs with acceptable laboratory operating costs (Garg and Dasouki, 2006; Levy, 2010; Ombrone et al., 2016). MS/MS was initially implemented in NBS to detect amino acid, organic acid, and fatty acid oxidation disorders in Shanghai in 2004 (Deng et al., 2021). However, the expanded NBS program was initiated in 2015 in Huaihua, Hunan Province.

Importantly, the incidence, disease spectrum, and genetic characteristics of IEMs are highly variable among different cities within Hunan Province in mainland China (Deng et al., 2021; Li et al., 2022; Zhou et al., 2022). Moreover, the spectrum and incidence of IEMs can vary among ethnic groups (Loeber et al., 2021). Therefore, considering that Huaihua is a multi-ethnic city adjacent to southwestern regions (National Bureau of Statistic, 2020), with ethnic minorities comprising approximately 40% of the total population, herein, we sought to report, for the first time, 7-year data from NBS incorporating MS/MS. In this way, we have analyzed the IEM incidence, disease spectrum, and genetic characteristics within the ethnically diverse Huaihua population. There have been no reports on the incidence of IEMs in Huaihua area. Our findings will lay the foundation for further research on the incidence of IEMs of each ethnic group in Hunan Province and Southwest China.

## 2 Materials and methods

### 2.1 Object

A total of 206,977 newborns with self-reported ethnicity born in Huaihua City, Hunan Province, from January 2015 to December 2021, were screened for various IEMs. After obtaining written informed consent from the parents, blood samples were collected and tested in the laboratory. This study protocol was approved by the Ethics Committee of the Maternal and Child Health Hospital of Huaihua City.

### 2.2 Blood sample collection

All procedures followed the current technical criteria of NBS established by the Ministry of Health of China (Ministry of Health of China, 2010). The infants had received adequate feeding before samples were collected between two and 7 days after birth. Capillary blood samples were taken via heel stick and spotted onto filter paper (S&S 903) with a minimum of four spots repeatedly dripped without squeezing and then air-dried on blood collection cards. The samples were delivered to the screening laboratory within five business days for MS/MS-based NBS.

### 2.3 Newborn screening

**FIGURE 1:**
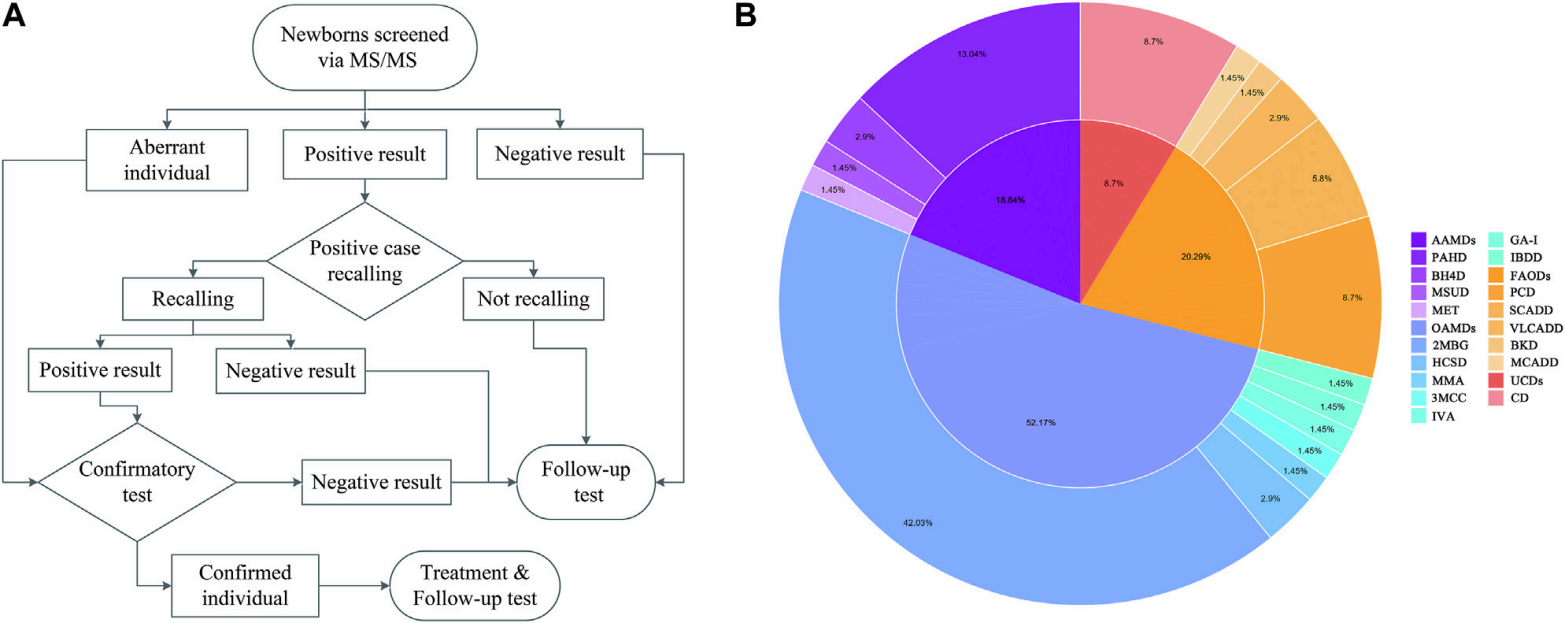
Flowchart of expanded newborn screening for inborn errors of metabolism (IEM) and genetic analysis of patients **(A)**. Proportion of IEMs in Huaihua area from 2015 to 2021 **(B)**.

Dried blood spots (DBSs) were obtained utilizing an automatic hole puncher and treated with amino acid standards and acylcarnitine. The samples were processed using the Neobase™ Non-derivative kit (PerkinElmer, Turku, Finland) and tandem mass spectrometry analysis system (Waters-ACQUITY TQD). After passing quality control, 86 analytes (Table 1) were analyzed, comprising 11 amino acids, one ketone, and 31 acylcarnitines, as well as their ratios. The MS/MS system established biological reference intervals based on 0.5 and 99.5 percentiles of blood amino acid and acylcarnitine levels in 3,000 healthy newborns (Wang, 2019). It sets disease screening indexes and positive cut-off values according to the disease characteristics. With the increase in the sample size, the system was revised yearly to minimize false positives and negatives. The flowchart is shown in Figure 1A.

**TABLE 1:** The 86 indicators of newborn screening.

No.	Screening indicators	Abbreviations	Cut-off values (μmol/L)	*N*	Ratios	Cut-off values
1	Free carnitine	C0	9.8–55	44	VAL/PHE	1.3–4.5
2	Acetylcarnitine	C2	3–45	45	TYR/PHE	0.6–4.62
3	Propionylcarnitine	C3	0–4.5	46	TYR/CIT	1.8–29
4	Malonylcarnitine+3-hydroxy butyrylcarnitine	C3DC + C4OH	0–0.37	47	SA/PHE	0–0.03
5	Butyrylcarnitine	C4	0.07–0.5	48	PRO/PHE	1.7–9.3
6	Methylmalonylcarnitine+3-hydroxy isovalerylcarnitine	C4DC + C5OH	0–0.4	49	PHE/TYR	0.15–1.38
7	Isovalerylcarnitine	C5	0.01–0.3	50	PHE/(C3+C16)	4–35
8	Tiglylcarnitine	C5:1	0–0.02	51	ORN/CIT	2.5–16
9	Glutarylcarnitine+3-hydroxy hexanoylcarnitine	C5DC + C6OH	0–0.23	52	MET/PHE	0.06–0.71
10	Hexanoylcarnitine	C6	0–0.1	53	MET/CIT	0.31–3.8
11	Adipylcarnitine	C6DC	0.03–0.25	54	GLY/PHE	3.5–16
12	Octanoylcarnitine	C8	0.01–0.15	55	CIT/PHE	0.11–0.7
13	Octenoylcarnitine	C8:1	0.04–0.4	56	CIT/ARG	0.4–17
14	Decenoylcarnitine	C10	0.02–0.22	57	C8/C2	0–0.01
15	Decanoylcarnitine	C10:1	0.02–0.14	58	C8/C10	0.4–1.5
16	Decadienoylcarnitine	C10:2	0–0.07	59	C5/C3	0.02–0.4
17	Dodecanoylcarnitine	C12	0.01–0.3	60	C5/C2	0–0.04
18	Dodecenoylcarnitine	C12:1	0–0.28	61	C5/C0	0–0.01
19	Myristoylcarnitine	C14	0.04–0.4	62	C4/C3	0.04–0.34
20	Myristoleylcarnitine	C14:1	0.03–0.3	63	C4/C2	0–0.04
21	Tetradecadienoylcarnitine	C14:2	0–0.05	64	C3/MET	0.02–0.38
22	3-hydroxy myristoylcarnitine	C14OH	0–0.04	65	C3/C2	0.03–0.26
23	Palmitoylcarnitine	C16	0.5–6.84	66	C3/C0	0.01–0.2
24	Hexadecenoylcarnitine	C16:1	0.03–0.5	67	C16OH/C16	0–0.02
25	3-hydroxy palmitoylcarnitine	C16OH	0–0.05	68	C16/C3	0.4–5.5
26	3-hydroxy palmitoleylcarnitine	C16:1OH	0–0.09	69	C14:1/C2	0–0.02
27	Octadecanoylcarnitine	C18	0.2–1.92	70	C14:1/C16	0.01–0.08
28	Octadecenoylcarnitine	C18:1	0.4–3.4	71	C14/C3	0.02–0.47
29	Linoleylcarnitine	C18:2	0.05–0.77	72	C10/C3	0.01–0.27
30	3-hydroxy octadecanoylcarnitine	C18OH	0–0.03	73	C0/(C16 + C18)	2.2–24
31	3-hydroxy octadecenoylcarnitine	C18:1OH	0–0.05	74	ARG/PHE	0.01–0.7
32	Alanine	ALA	120–730	75	ARG/ORN	0.01–0.41
33	Arginine	ARG	1–43	76	ALA/CIT	9.5–65
34	Citrulline	CIT	6–27	77	(LEU + ILE + PRO-OH)/TYR	0.6–5.6
35	Glycine	GLY	178–980	78	(LEU + ILE + PRO-OH)/PHE	1.56–4.9
36	Leucine + isoleucine + hydroxyproline	LEU + ILE + PRO-OH	70–280	79	(C5DC + C6OH)/C16	0.01–0.15
37	Methionine	MET	7–37	80	(C5DC + C6OH)/(C4DC + C5OH)	0.15–1.27
38	Ornithine	ORN	35–250	81	(C5DC + C6OH)/(C3DC + C4OH)	0.2–2
39	Phenylalanine	PHE	23–95	82	(C4DC + C5OH)/C8	1.23–12
40	Proline	PRO	90–390	83	(C4DC + C5OH)/C0	0–0.02
41	Succinylacetone	SA	0–1.3	84	(C3DC + C4OH)/C10	0.4–4.5
42	Tyrosine	TYR	25–250	85	(C16 + C18:1)/C2	0.13–1
43	Valine	VAL	51–220	86	(C0+C2+C3+C16 + C18:1 + C18)/CIT	1.43–12

### 2.4 Positive neonatal screening results

Newborns who were positive based on primary screening were referred for repeat tests. If the screening result continued to increase without subsequently decreasing, arrangements were made for the infant to be evaluated at special medical centers. However, if the infant exhibited aberrant symptoms, they were diagnosed, and treatment was immediately initiated. There are 48 types of IEM conditions included in the NBS. The IEM specialist evaluated the positive diagnosis of the IEMs based on the GC-MS, MALDI-TOF MS, next-generation sequencing (NGS) results (Biosan, Hangzhou, China), and designated analytes, ratios, and cut-off values. The positive rules of IEMs are shown in Table 2.

**TABLE 2:** Positive rules in expanded newborn screening panel.

Disorders (OMIM code)	Positive rules 1	Positive rules 2	Positive rules 3	Positive rules 4
2,4-Dienoyl-CoA reductase deficiency (#616,034)	C10:2 > 0.1 μmol/L			
Medium chain acyl CoA dehydrogenase deficiency (#201,450)	C6>0.1 μmol/L, C8>0.15 μmol/L, C8/C2>0.01, (C4DC + C5OH)/C8<1.23			
Short chain acyl CoA dehydrogenase deficiency (#201,470)	C4>0.5 μmol/L, C4/C2>0.04	C4>1 μmol/L		
Very long chain acyl CoA dehydrogenase deficiency (#201,475)	C14:1 > 0.3 μmol/L, C14:1/C16 > 0.08, C14:1/C2>0.02	C14:1 > 0.5 μmol/L		
Argininemia (#207,800)	ARG/PHE>0.7, ARG/ORN>0.41, ARG>43 μmol/L, CIT/ARG<0.4	ARG>80 μmol/L		
Holocarboxylase synthetase deficiency (#253, 270), 3-methylglutaconyl CoA hydratase deficiency (#250,950), 3-methylcrotonyl CoA carboxylase deficiency (#210,200 and #210,210), 3-hydroxy-3-methylglutaryl-CoA lyase deficiency (#246,450)	C4DC + C5OH > 0.4, (C4DC + C5OH)/C0>0.02	C4DC + C5OH > 0.6		
Beta-ketothiolase deficiency (#203,750)	C3DC + C4OH > 0.37, C5:1 > 0.01 μmol/L, C4DC + C5OH > 0.31, (C4DC + C5OH)/C0>0.02			
Maple syrup urine disease (#248,600)	LEU + ILE + PRO-OH>270, (LEU + ILE + PRO-OH)/PHE>4.9, VAL>210 μmol/L	LEU + ILE + PRO-OH>500 μmol/L		
Glutaric acidemia type Ⅰ (#231,670)	C5DC + C6OH > 0.23, (C5DC + C6OH)/(C3DC + C4OH) > 2, (C5DC + C6OH)/(C4DC + C5OH) > 1.27	C5DC + C6OH > 0.3 μmol/L		
Citrullinemia type Ⅰ (#215,700), Citrin deficiency (Citrullinemia (#605,814 and #603,471), Argininosuccinic aciduira (#207,900)	CIT>27 μmol/L, ALA/CIT<9.5	CIT>60 μmol/L		
Hyperornithinemia-hyperammonemia-homocitrullinuria syndrome (#238,970)	ORN>250 μmol/L, ORN/CIT>16			
Long chain 3-hydroxy acyl-CoA dehydrogenase deficiency (#609,016), trifunctional protein deficiency (#609,015)	C16OH > 0.04 μmol/L, C16OH/C16 > 0.01, C18:1OH > 0.04 μmol/L, C18OH > 0.02 μmol/L			
Homocystinuria (#236,200), Hypermethioninemia (#250,850)	MET>37 μmol/L, MET/PHE>0.71	MET>70 μmol/L		
Malonic acidemia (#248,360)	C3DC + C4OH > 0.37 μmol/L, (C3DC + C4OH)/C10 > 4.5			
Nonketotic hyperglycinemia (#617, 301, #605,899)	GLY>980 μmol/L			
Tyrosinemia type Ⅱ (#276,600), Tyrosinemia type Ⅲ (#276,710)	TYR>250 μmol/L, (LEU + ILE + PRO-OH)/TYR<0.6, TYR/PHE>4.62	TYR>400 μmol/L		
Carnitine palmitoyltransferase I deficiency (#255,120)	C0/(C16 + C18)>24, C0>55 μmol/L, (C16 + C18:1)/C2<0.13	C0>100 μmol/L		
Tyrosinemia type I (#276,700)	SA>1.3 μmol/L, SA/PHE>0.03	SA>2 μmol/L		
Multiple acyl-CoA dehydrogenase deficiency (#231,680)	C5>0.3 μmol/L, C4>0.5 μmol/L			
Phenylalanine hydroxylase deficiency (#261,600), Tetrahydrobiopterin deficiency (#233,910, #261,640, #612,716, #264,070, and #261,630)	PHE>95 μmol/L, PHE/TYR>1.2	PHE/TYR ≥ 2.4	PHE>120 μmol/L	
Primary carnitine deficiency (#212,140)	C0<9 μmol/L	C0<9.8 μmol/L, (C0+C2+C3+C16 + C18 + C18:1)/CIT<1.5	C0<9.8 μmol/L, PHE/(C3+C16)>30	
Carbamoylphosphate synthetase I deficiency (#237,300), ornithine aminotransferase deficiency (#258,870), ornithine transcarbamylase deficiency (#311,250)	CIT<6 μmol/L, CIT/PHE<0.11	CIT<4.5 μmol/L		
Red cell pyruvate kinase deficiency (#266,200)	CIT>27 μmol/L, MET/CIT<0.31			
Hyperprolinemia (#239,500)	PRO>460 μmol/L			
Isobutyryl-CoA dehydrogenase deficiency (#611,283), ethylmalonic encephalopathy (#602,473)	C4/C3>0.34, C4/C2>0.04	C4>0.7 μmol/L		
Methylmalonic acidemia (#251,000, #277,400, #277,410, #251,100, #251,110, #277,380, #309,541, #613, 646, #614, 265 and #614, 857), propionic acidemia (#606,054)	C3>4.5 μmol/L, C3/C2>0.2	C3/C2>0.32	C3>6.5 μmol/L	C3/C0>0.4
Carnitine palmitoyltransferase Ⅱ deficiency (#255,110, #608,836, #600,649), Carnitine/acylcarnitine translocase deficiency (#212,138)	(C16 + C18:1)/C2>0.5, C16 > 6.84 μmol/L, C0/(C16 + C18)<2.4			
Isovaleric acidemia (#243,500), 2-methylbutryl CoA dehydrogenase deficiency (#610,006)	C5>0.3 μmol/L, C5/C0>0.01	C5>0.8 μmol/L		

### 2.5 Gene analysis

Amplicon target capture and NGS technology were applied to samples. Total genomic DNA was extracted from dried blood samples with the Qiagen Blood DNA Mini Kit (Qiagen, Düsseldorf, Germany) according to the manufacturer’s instructions. They were then treated with capture probes (Agilent, Santa Clara, CA, USA) for 86 genes, including *PAH*, *PTS*, *PCBD1*, *QDPR*, *SPR*, and *GCH1*. The library underwent quality control by determining the fragment length and size distribution using a Qubit and 2,100 Bioanalyzer (Agilent High Sensitivity DNA Kit, Agilent). Subsequently, a quantitative kit (Illumina DNA Standards and Primer Premix Kit, Kapa) was used to quantify the library and determine the sample size. Finally, sequencing was performed with the HiSeq 2,500 (Illumina) platform. The ACMG (Richards et al., 2015) guidelines were used to interpret the pathogenicity of variants, and Sanger sequencing was used for locus verification of the discovered variants.

### 2.6 Statistical analysis

Statistical analyses were performed using SPSS19.0. The chi-square test was performed to compare the patients diagnosed with each disorder. Differences in the screening data were compared by analysis of variance (One-way ANOVA). Statistical significance was set at *p* < 0.05.

## 3 Results

### 3.1 Neonatal screening

A total of 206,977 newborns were screened using MS/MS; among them, the gender index comprised 110,710 males, 96,106 females, and 92 babies with no record; the average age at first screening was ∼7.09 days. The proportions of infants born preterm or with low birthweight were 4.25% (8,805/206,977) and 3.33% (6,892/206,977), respectively; newborn twins and triplets were 0.57% (1,190/206977), and 98.42% (203708/206977) of the infants who completed screening in the Huaihua area. No significant differences were observed in any demographic characteristics between normal newborns and IEM patients (Table 3).

**TABLE 3:** Characteristics of newborns screened by expanded newborn screening program.

Category	Newborns without targeted IEMs	Patients with IEMs	*p*-Value
Age at initial testing (days, mean ± SD)	7.09 ± 3.49	7.4 ± 3.53	0.283
Number	206,908	69	
Sex			
Male	110,710	39	0.87
Female	96,106	30	
No record	92	0	
Gestational age (weeks)			
<32	458	0	0.74
32–36	8,347	4	
>37	184,268	62	
No record	13,835	3	
Birth weight (g)			
<1,500	366	0	0.861
1,500–1,999	1,118	0	
2,000–2,499	5,408	3	
>2,500	190,458	63	
No record	9,558	3	
Number of fetuses			
Singleton	205,715	68	0.627
Twins	1,184	1	
Triplet	9	0	
Register region			
Huaihua	203,749	69	0.586
Others	1,991	0	
No record	1,168	0	

A total of 5,578 cases were screened as positive, accounting for a 2.69% positive rate upon initial screening. Of these, only 4,085 (73.23%) babies were recalled for repeat tests because we unable to contact with the parents and some parents declined to retest. 297 infants were referred to a diagnostic test. Ultimately, 69 IEM cases were diagnosed, yielding an estimated overall incidence of 1:3,000. As no false-negatives were detected in our 7-year data, our IEMs diagnostic testing overall sensitivity was 100% and specificity was 99.90%. The 69 patients were divided into four classes of IEMs: amino acid disorders (AAMDs), organic acid disorders (OAMDs), fatty acid oxidation disorders (FAODs), and urea cycle disorders (UCDs), with incidence rates of 1:15,921, 1:5,749, 1:14,784, and 1:34,496, respectively. As an ethnically diverse city, the IEM incidence of the minority ethnic group was 1:1852, while that of the Han ethnic group was 1:4,741. Among the minority ethnic groups, the incidence rate of the Dong, Miao, Tujia, and Yao ethnic groups was 1:2,117, 1:1,612, 1:2,005, and 1:1,769, respectively (Table 4).

**TABLE 4:** Newborn screening of the Huaihua area from 2015 to 2021.

Year	Number of screenings	Suspected positive cases	Confirmed cases	Frequency
2015	14,945	312	2	1:7,473
2016	28,020	528	7	1:4,003
2017	35,391	662	12	1:2,949
2018	36,167	952	6	1:6,028
2019	34,712	1,043	16	1:2,170
2020	30,378	1,104	10	1:3,038
2021	27,364	977	16	1:1,710
Total	206,977	5,578	69	1:3,000

### 3.2 Incidence

Four types of AAMD were detected in the Han population (1:21,334), the incidence of which was significantly lower than that of the Miao population (1:10,746), Dong population (1:17,798), Tujia population (1:6,015), and Yao population (1:3,538). Phenylalanine hydroxylase deficiency (PAHD, 1:22,997) was the most common, accounting for 69.23% of AAMDs. Seven types of OAMDs were detected in Huaihua City, the highest incidence was in 2-methylbutyryl glycinuria (2MBG, 1:7,137), accounting for 80.56% of OAMDs. The Dong population (1:3,000) had the highest incidence of 2MBG, followed by the Tujia population (1:3,008), Yao population (1:3,538), Miao population (1:5,373) and Han population (1:16,001). Among the five types of FAODs, primary carnitine deficiency (PCD, 1:34,496) had the highest incidence, accounting for 42.86% of FAODs. Meanwhile, medium-chain acyl-CoA dehydrogenase deficiency (MCADD, 1:128,004) was only identified in the Han population; very long-chain acyl-CoA dehydrogenase deficiency (VLCADD, 1:16,119) and beta-ketothiolase deficiency (BKD, 1:32,237) were only identified in the Miao population. The above data are presented in Table 5, and the IEM proportions are illustrated in Figure 1B.

**TABLE 5:** Proportion and incidence of IEMs in different ethnic groups in Huaihua. *Incidence*a, the incidence of each IEM without the classification of diverse ethnic group in Huaihua; *Incidence*b, the incidence of each ethnic group in Huaihua.

Disorder (OMIM code)	Han	Miao	Dong	Tujia	Yao	Total	Incidencea
AAMDs							
PAHD (#261600)	2	3	2	1	1	9	1:22,997
BH4D (#233910, #261640, #612716, #264070, and #261630)	2					2	1:103,489
MSUD (#248600)	1					1	1:206,977
MET (#250850)	1					1	1:206,977
OAMDs							
2MBG (#610006)	8	6	12	2	1	29	1:7,137
MMA (#251000)	2					2	1:103,489
3MCC (#210200 and #210210)	1					1	1:206,977
HCSD (#253270)	1					1	1:206,977
IVA (#243500)		1				1	1:206,977
GA-I (#231670)	1					1	1:206,977
IBDD (#611283)	1					1	1:206,977
FAODs							
PCD (#212140)	2	3	1			6	1:34,496
SCADD (#201470)	1	1	2			4	1:51,744
VLCADD (#201475)		2				2	1:103,489
BKD (#203750)		1				1	1:206,977
MCADD (#201450)	1					1	1:206,977
UCDs							
CD (#605814 and #603471)	3	3				6	1:34,496
Total						69	1:3,000
Incidenceb	1:4,741	1:1,612	1:2,117	1:2,005	1:1,769		

### 3.3 Genetic analyses

Genetic variant identified in the ethnic groups were deposited in ClinVar:

https://www.ncbi.nlm.nih.gov/clinvar/?term=SUB14302371. The variant c.728G>A was the most commonly detected in *PAH*, accounting for 27.78% of all *PAH* variants. One patient from the Han population with MSUD was found to carry two variants, c.632C>T (Like Pathogenic) and c.673C>T (Uncertain Significant). Moreover, one patient with MET carried a single pathogenic variant c.791G>A. The 29 patients with 2MBG carried five variants; c.1165A>G was the most commonly identified in the *ACADSB* gene. Interestingly, the c.1165A>G variant was detected in all five ethnic groups, and 21/29 patients were identified as homozygotes.

**FIGURE 2:**
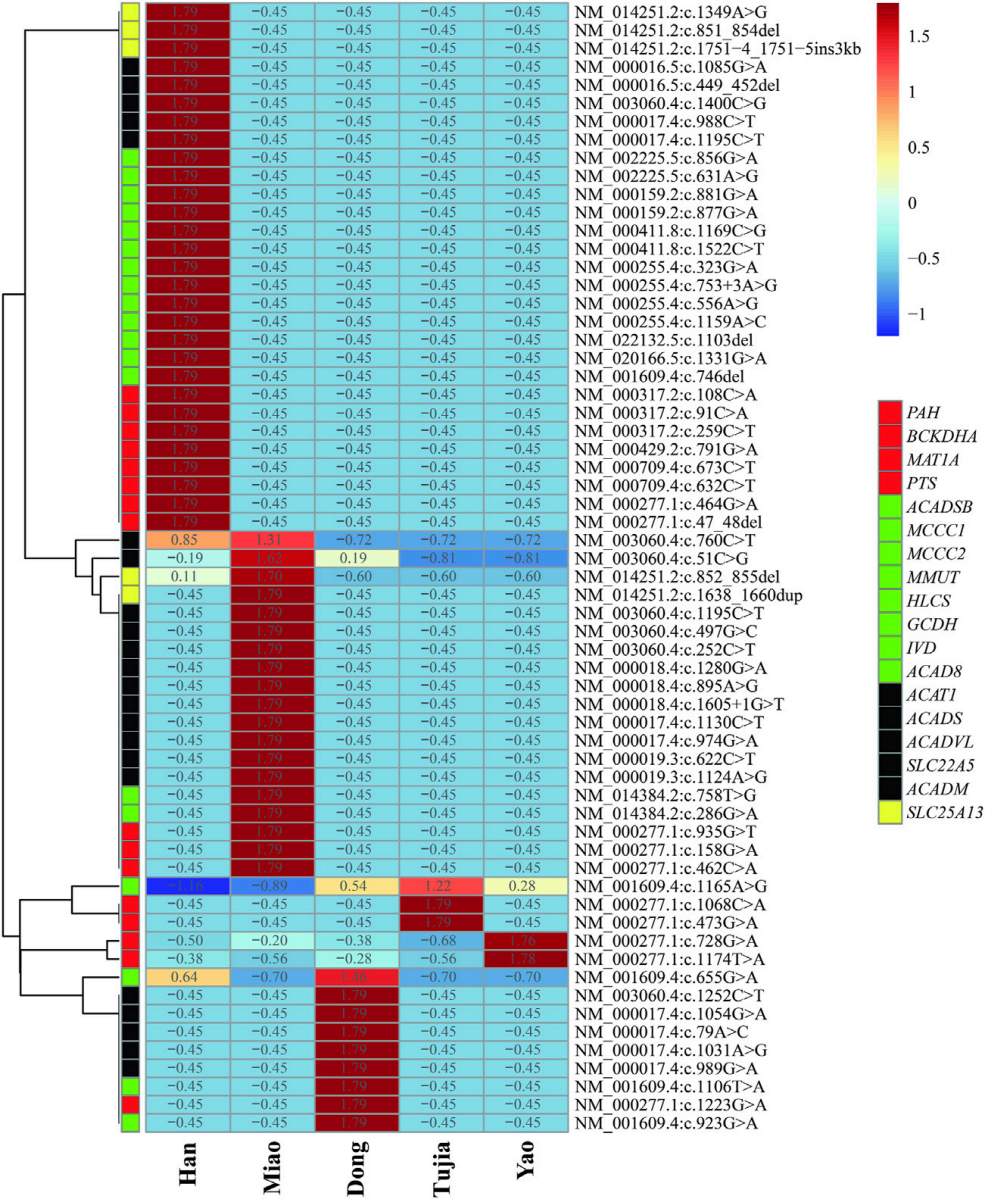
Heatmap of inborn errors of metabolisms (IEM) variants frequency distributions in five ehtnic groups in Huaihua area; The blue to the red of the upper legend indicates the lowest variants frequency to the highest variants frequency; The red, green, black and yellow of the lower legend indicates the gene of AAMDs, OAMDs, FAMDs, and UCDs, respectively.

In the six patients from the Han population with OAMD, c.1522C>T and c.1169C>G were detected in the *HLCS* gene of HCSD; c.1331G>A was detected in *MCCC1*, while c.1103del was detected in *MCCC2* of 3MCC. Six patients with PCD (two Han, three Miao, and one Dong) carried seven variants; The c.51C>G variant was the most frequently detected in the *SLC22A5* gene and was a hotspot variant detected in three main ethnic groups. Four SCADD patients (one Han, one Miao, and two Dong) carried eight variants of the *ACADS* gene. Two Miao patients were identified with VLCADD; c.1605 + 1G>T, c.895A>G, and c.1280G>A were detected in the *ACADVL* gene. The six patients with CD (three Han and three Miao) carried five variants; c.852_855del was the most frequently detected in the *SLC25A13* gene, occurring in 58.33% of *SLC25A13* gene variants. Three out of six patients with the c.852_855del variant were identified as homozygotes. All the variants are presented in Table 6. There were huge differences in IEM variant spectrum in different ethnic groups which are shown in Figure 2.

**TABLE 6:** Variants detected in five ethnic groups patients with IEMs identified by expanded newborn screening.

Conditions (OMIM number)	Gene (OMIM number)	Variant_HGVS	Amino acid variant	Pathogenic	Han	Miao	Dong	Tujia	Yao	Variant number	Relative frequency (%)	Accounting for total variants (%)
PAHD (#261600)	*PAH* (*612349)									18		13.14
		NM_000277.1:c.728G>A	p. (R243Q)	P	1	2	1		1	5	27.78	3.65
		NM_000277.1:c.1174T>A	p. (F392I)	LP	1		1		1	3	16.67	2.19
		NM_000277.1:c.158G>A	p. (R53H)	VUS		2				2	11.11	1.46
		NM_000277.1:c.1223G>A	p. (R408Q)	P			2			2	11.11	1.46
		NM_000277.1:c.1068C>A	p. (Y356*)	P				1		1	5.56	0.73
		NM_000277.1:c.462C>A	p. (Y154*)	P		1				1	5.56	0.73
		NM_000277.1:c.464G>A	p. (R155H)	P	1					1	5.56	0.73
		NM_000277.1:c.47_48del	p. (S16*)	P	1					1	5.56	0.73
		NM_000277.1:c.473G>A	p. (R158Q)	P				1		1	5.56	0.73
		NM_000277.1:c.935G>T	p. (G312V)	P		1				1	5.56	0.73
MSUD (#248600)	*BCKDHA* (*608348)									2		1.46
		NM_000709.4:c.632C>T	p. (T211M)	LP	1					1	50.00	0.73
		NM_000709.4:c.673C>T	p. (R225W)	VUS	1					1	50.00	0.73
MET (#250850)	*MAT1A* (*610550)									1		0.73
		NM_000429.2:c.791G>A	p. (R264H)	P	1					1	100.00	0.73
BH4D (#233910,#261640, #612716,#264070, and #261630)	*PTS* (*612719)									4		2.92
		NM_000317.2:c.259C>T	p. (P87S)	P	2					2	50.00	1.46
		NM_000317.2:c.91C>A	p. (L31I)	VUS	1					1	25.00	0.73
		NM_000317.2:c.108C>A	p. (N36K)	LP	1					1	25.00	0.73
2MBG (#610006)	*ACADSB* (*600301)									57		41.61
		NM_001609.4:c.1165A>G	p. (M389V)	P	13	12	18	4	2	49	85.96	35.77
		NM_001609.4:c.655G>A	p. (V219M)	P	2		2			4	7.02	2.92
		NM_001609.4:c.923G>A	p. (C308Y)	P			2			2	3.51	1.46
		NM_001609.4:c.746del	p. (P249Lfs*15)	LP	1					1	1.75	0.73
		NM_001609.4:c.1106T>A	p. (M369K)	VUS			1			1	1.75	0.73
3MCC (#210200 and #210210)										2		1.46
	*MCCC1* (*609010)	NM_020166.5:c.1331G>A	p. (R444H)	VUS	1					1	50.00	0.73
	*MCCC2* (*609014)	NM_022132.5:c.1103del	p. (G368Vfs*70)	P	1					1	50.00	0.73
MMA (#251000)	*MMUT* (*609058)									4		2.92
		NM_000255.4:c.1159A>C	p. (T387P)	P	1					1	25.00	0.73
		NM_000255.4:c.556A>G	p. (M186V)	LP	1					1	25.00	0.73
		NM_000255.4:c.753 + 3A>G	—	LP	1					1	25.00	0.73
		NM_000255.4:c.323G>A	p. (R108H)	P	1					1	25.00	0.73
HCSD (#253270)	*HLCS* (*609018)									2		1.46
		NM_000411.8:c.1522C>T	p. (R508W)	P	1					1	50.00	0.73
		NM_000411.8:c.1169C>G	p. (S390*)	LP	1					1	50.00	0.73
GA-I (#231670)	*GCDH* (*608801)									2		1.46
		NM_000159.2:c.877G>A	p. (A293T)	P	1					1	50.00	0.73
		NM_000159.2:c.881G>A	p. (R294Q)	LP	1					1	50.00	0.73
IVA (#243500)	*IVD* (*607036)									2		1.46
		NM_014384.2:c.286G>A	p. (96S)	LP		1				1	50.00	0.73
		NM_014384.2:c.758T>G	p. (V253G)	VUS		1				1	50.00	0.73
IBDD (#611283)	*ACAD8* (*604773)									2		1.46
		NM_002225.5:c.631A>G	p. (T211A)	P	1					1	50.00	0.73
		NM_002225.5:c.856G>A	p. (V286M)	LP	1					1	50.00	0.73
BKD (#203750)	*ACAT1* (*607809)									2		1.46
		NM_000019.3:c.1124A>G	p. (N375S)	P		1				1	50.00	0.73
		NM_000019.3:c.622C>T	p. (R208X)	P		1				1	50.00	0.73
SCADD (#201470)	*ACADS* (*606885)									8		5.84
		NM_000017.4:c.974G>A	p. (R325Q)	LP		1				1	12.50	0.73
		NM_000017.4:c.1130C>T	p. (P377L)	P		1				1	12.50	0.73
		NM_000017.4:c.989G>A	p. (R330H)	P			1			1	12.50	0.73
		NM_000017.4:c.1031A>G	p. (E344G)	P			1			1	12.50	0.73
		NM_000017.4:c.1195C>T	p. (R399W)	P	1					1	12.50	0.73
		NM_000017.4:c.988C>T	p. (R330C)	LP	1					1	12.50	0.73
		NM_000017.4:c.79A>C	p. (T27P)	VUS			1			1	12.50	0.73
		NM_000017.4:c.1054G>A	p. (A352T)	P			1			1	12.50	0.73
VLCADD (#201475)	*ACADVL* (*609575)									4		2.92
		NM_000018.4:c.1605 + 1G>T	—	P		2				2	50.00	1.46
		NM_000018.4:c.895A>G	p. (K299E)	VUS		1				1	25.00	0.73
		NM_000018.4:c.1280G>A	p. (W427*)	P		1				1	25.00	0.73
PCD (#212140)	*SLC22A5* (*603377)									13		9.49
		NM_003060.4:c.51C>G	p. (F17L)	P	1	3	1			5	38.46	3.65
		NM_003060.4:c.760C>T	p. (R254*)	P	1	1				2	15.38	1.46
		NM_003060.4:c.1400C>G	p. (S467C)	P	2					2	15.38	1.46
		NM_003060.4:c.252C>T	p. (Y84Y)	VUS		1				1	7.69	0.73
		NM_003060.4:c.497G>C	p. (R166T)	VUS		1				1	7.69	0.73
		NM_003060.4:c.1252C>T	p. (Q418*)	P			1			1	7.69	0.73
		NM_003060.4:c.1195C>T	p. (R399W)	P		1				1	7.69	0.73
MCADD (#201450)	*ACADM* (*607008)									2		1.46
		NM_000016.5:c.449_452del	p. (T150Rfs*4)	P	1					1	50.00	0.73
		NM_000016.5:c.1085G>A	p. (G362E)	P	1					1	50.00	0.73
CD (#605814 and #603471)	*SLC25A13* (*603859)									12		8.76
		NM_014251.2:c.852_855del	p. (M285Pfs*2)	P	2	5				7	58.33	5.11
		NM_014251.2:c.1751-4_1751-5ins3kb	p. (A584Vfs*2)	P	2					2	16.67	1.46
		NM_014251.2:c.851_854del	p. (R284_M285*fs)	LP	1					1	8.33	0.73
		NM_014251.2:c.1638_1660dup	p. (A554Gfs*17)	P		1				1	8.33	0.73
		NM_014251.2:c.1349A>G	p. (E450G)	VUS	1					1	8.33	0.73

## 4 Discussion

In our study, the prevalence of screened IEMs in Huaihua (1:3,000) far exceeded that in Changsha (1:4,237) (Li et al., 2022) and Shaoyang (1:4,115) (Zhou et al., 2022) (both in Hunan Province), and was higher than those in eastern cities, including Suzhou (1:3,163) (Wang et al., 2019) and Shanghai (1:5,800) (Gu et al., 2008), similar to districts in southern China, such as Quanzhou (1:2,804) (Lin et al., 2019) and Guangzhou (1:2,451) (Tang et al., 2021), and lower than that in the northern cities of Xi’an (1:1,898) (Zhang et al., 2021) and Jining (1:1,941) (Yang et al., 2020).

The most common IEM in Huaihua was 2MBG, detected in Han and ethnic minorities. Meanwhile, the US Wisconsin (Van Calcar et al., 2013) screening results showed that 90 of the 92 confirmed 2MBG cases belonged to the Miao ethnic group, which agreed with our findings, as the incidence rate of IEM in the Miao ethnic group was markedly higher than that of the Han nationality in Huaihua (2.9 times). The first cases of 2MBG with the identification of hypoevolutism were reported in 2000 (Andresen et al., 2000). Symptomatic patients present with developmental abnormalities, intellectual disturbance, seizures, muscular atrophy, and failure to thrive (Gibson et al., 1999; Sass et al., 2008; Alfardan et al., 2010). Genetic analyses of these cases revealed that all patients carried the same sole homozygous variant c.1165A>G (Van Calcar et al., 2013). Meanwhile, the common variants among Chinese populations are c.1165A>G and c.275C>G (Lin et al., 2019; Wang et al., 2019). In the current study, we reported 29 confirmed cases, in which the c.1165A>G variant was the most prevalent (85.96%), followed by c.655G>A (7.02%). Moreover, the proportion of homozygotes reached 72.41% (21/29), with all homozygote variants determined to be c.1165A>G. Hence, together with the previously published studies, these results indicate that c.1165A>G is the most common variant associated with 2MBG.

Interestingly, we found that the incidence of IEMs in the Huaihua minority ethnic groups (1:1,852) was more than two times that of the Han ethnic group (1:4,741). Although few studies have reported on the prevalence of genetic metabolic diseases among Chinese ethnic minorities, in 2020, a 4-year study in a multi-ethnic region of China revealed that the Hui nationality (Mao et al., 2020), as one of the minorities in China, has a significantly higher incidence rate of IEMs than the Han, which is consistent with our findings. Moreover, Zhao et al. (2019) reported that the total carrier frequency of 11 genetic diseases varies among Chinese ethnic groups, including Miao (27.15%) and Han (25.28%). However, PAHD is the only disease that was evaluated by Zhao et al. (2019) and the current study; the carrier frequency of Miao (14.32%) was significantly higher than that of Han (3.30%), Dong (5.60%), and Tujia (4.91%). Moreover, we found that the incidence rate of PAHD was the highest in the Yao ethnic group (1:3,538), while the prevalence in Tujia (1:6,015) was higher than in Miao (1:10,746). Given that there do not appear to be other reports on the incidence rate of IEMs within the Dong, Tujia, and Yao ethnic minorities in China, the current study serves as the first report on the disease spectrum and gene variant spectrum of IEMs among these three ethnic minorities.

In addition, nine IEM types were detected only in the Han nationality population, namely, BH4D, MSUD, MET, MMA, 3MCC, HCSD, GA-I, IBDD, and MCADD, whereas BKD, VLCADD, and IVA were only found in the minority nationality population in Huaihua. BKD was first described in 1971 and is encoded by *ACAT1* presenting as severe metabolic acidosis, hypoglycemia, ketonuria, and abnormal liver function (Mrázová et al., 2005; Grünert et al., 2017; Fukao et al., 2019). However, BKD is rare, as reported for Tunisia (Monastiri et al., 1999) and China (1:136,059-1:2,366,555) (Deng et al., 2021; Tang et al., 2021). In the current study, one case of BKD was detected in a Miao patient with an incidence rate of 1:206,977, which is slightly higher than the average incidence. Moreover, we detected two pathogenic variants (c.1124A>G and c.622C>T) in *ACAT1*. Reports from Vietnam (Nguyen et al., 2017) suggest that c.622C>T and C.1006-1G>C are the most common variants, while C.622C > T has been detected in the Kinh ethnic population.

VLCADD is encoded by *ACADVL* and is characterized by infections with fever, multi-organ failure, a low mortality rate, and myoglobinuria after exercise (Pena et al., 2016; Olsson et al., 2022; Osawa et al., 2022). Our study detected two cases of VLCADD in Miao patients (1:103,489). We detected three variants in *ACADVL* (c.1605 + 1G>T, c.895A>G, and c.1280G>A), of which c.895A>G and c.1280G>A were heterozygous. Meanwhile, c.848T>C is reportedly a hotspot variant in VLCADD cases in the United States and Switzerland (Pena et al., 2016; Olsson et al., 2022), and c.1820G>C (p. (Cys607Ser)) (Osawa et al., 2022) is the hotspot variant in Japanese patients. However, we did not detect either of these variants in the Huaihua population. Moreover, c.1280G>A has been reported in Suzhou (Wang et al., 2019), Shaoyang (Zhou et al., 2022), and the Ningxia Hui nationality (Mao et al., 2020). Consequently, the hotspot variants in different countries, regions, and ethnic groups are diverse; however, c.1280G>A may represent the most common variant in most ethnic groups in China.

IVA, encoded by the *IVD* gene, is classified into two diverse phenotypes: the acute neonatal form causes metabolic acidosis, leading to lethargy, vomiting, coma, or death, while the chronic form typically has a late onset in childhood with an asymptomatic phenotype (Vockley and Ensenauer, 2006; Schlune et al., 2018; Ding et al., 2022). One Miao patient was identified with IVA with acute clinical symptoms; genetic analysis detected two variants (c.631A>G and c.856G>A).

Although ninety-five variants were identified in Huaihua, only seven were shared by Han, Miao, Dong, Tujia, and Yao ethnic groups. Hence, different ethnic groups carry unique variants. However, the different disease profiles among ethnic groups correspond with differences in variants. In fact, even in diseases jointly discovered in Han and ethnic minorities, the distribution of variants was inconsistent. More specifically, we detected five common conditions and 35 variant sites among the Han and ethnic minorities in Huaihua. The prevalence of IEMs in ethnic minorities (Miao, Dong, Tujia, Yao, et al.) with strong racial and regional characteristics was much higher than in the Han nationality. Consequently, each country or region with multiple ethnic groups should create a local disease spectrum standard of IEMs, according to the local demographic composition. Within the current study, the prevalence of IEMs in ethnic minorities (Miao, Dong, Tujia, Yao, et al.) with strong racial and regional characteristics was much higher than in the Han nationality. This might be owing to the unique marriage practices of the Chinese ethnic minorities resulting in the selection of partners within the same ethnic group. Moreover, most ethnic minority areas are in mountainous regions with limited transportation routes, encouraging the selection of partners from neighboring regions. However, these postulations require further verification by establishing the carrying rate of IEM-related pathogenic gene variants in the different ethnic minorities in Huaihua.

Certain limitations were noted in this study. First, owing to the insufficient sample size, more confirmed data is needed to supplement the genotypes of the Tujia and Yao ethnic groups. Second, there are many ethnic minorities in the Huaihua area (National Bureau of Statistic, 2020); they were not all represented in our study. Hence, additional data is needed to investigate the IEM spectrum and genotype of these ethnic minorities.

## 5 Conclusion

This study is the first to report the overall incidence of IEMs in the Huaihua area and the disease and gene variant spectrum of IEMs in the Miao, Dong, Tujia, and Yao ethnic groups. Importantly, the IEM incidence within the minority ethnic groups is markedly higher than among the Han nationality in Huaihua. This study serves as a theoretical reference for the screening and diagnosing of neonatal IEMs in the Huaihua area, and across China, especially the multi-ethnic areas. We proposed that IEMs in ethnic groups should be shown more solicitude because of their high incidence. Meanwhile, the identification of ethnic variants could impact future screening approaches such as newborn genetic screening, by designing panels based on ethnic variants database, which could reduce newborn screening costs and enhance screening coverage of IEMs.

## Data Availability

The datasets presented in this study can be found in online repositories. The names of the repository/repositories and accession number(s) can be found in the article/Supplementary material.

## Abbreviations

IEMs, Inborn errors of metabolism; 2MBG, 2-Methylbutyryl glycinuria; PAHD, Phenylalanine hydroxylase deficiency; NBS, Newborn screening; MS/MS, Tandem mass spectrometry; DBS, Dried blood spot; AAMDs, Amino acid disorders; OAMDs, Organic acid disorders; FAMDs, Fatty acid oxidation disorders; UCDs, Urea cycle disorders; MET, Hypermethioninemia; 3MCC, 3-methylcrotonyl-CoA carboxylase deficiency; SCADD, Short-chain acyl-CoA dehydrogenase deficiency; PCD, Primary carnitine deficiency; CD, Citrin deficiency; MSUD, Maple syrup urine disease; BH4D, Tetrahydrobiopterin deficiency; HCSD, Holocarboxylase synthetase deficiency; GA-I, Glutaric acidemia type Ⅰ; IVA, Isovaleric acidemia; BKD, Beta-ketothiolase deficiency; VLCADD, Very long-chain acyl-CoA dehydrogenase deficiency; MCADD, Medium-chain acyl-CoA dehydrogenase deficiency; MMA, Methylmalonic acidemia; IBDD, Isobutyryl-CoA Dehydrogenase Deficiency.
